# BuZhong YiQi Formula Alleviates Diabetes-Caused Hyposalivation by Activating Salivary Secretion Pathway in the Parotid and Submandibular Glands of Rats

**DOI:** 10.3390/ph18030377

**Published:** 2025-03-06

**Authors:** Ming-Yu Wang, Zhen-Ran Hu, Liang Wang, Xin-Xin Zeng, Xiang-Ke Li, Guo-Jun Fei, Jing-Li Zhang, Jing-Ru Chen, Ze-Min Yang

**Affiliations:** 1 Department of Biochemistry and Molecular Biology, School of Basic Medical Sciences, Guangdong Pharmaceutical University, Guangzhou 510006, China; wmy112222@163.com (M.-Y.W.); 2112255029@stu.gdpu.edu.cn (Z.-R.H.); liang7982023@163.com (L.W.); zengxinxin27@163.com (X.-X.Z.); kekeli123123@163.com (X.-K.L.); feiguojun2024@163.com (G.-J.F.); zjl17688633641@163.com (J.-L.Z.); chenjr0325@163.com (J.-R.C.); 2 Guangdong Provincial Key Laboratory of Pharmaceutical Bioactive Substances, School of Basic Medical Sciences, Guangdong Pharmaceutical University, Guangzhou 510006, China

**Keywords:** type 2 diabetes mellitus, BuZhong YiQi formula, hyposalivation, salivary secretion pathway, parotid gland, submandibular gland

## Abstract

**Background/Objectives**: BuZhong Yiqi Formula (BZYQF) has significant ameliorative effects on type 2 diabetes mellitus (T2DM). However, its efficacy in alleviating the hyposalivation caused by T2DM needs to be confirmed, and its mechanism is unclear. **Methods**: Network pharmacology and molecular docking were combined to analyze the molecular mechanism by which BZYQF alleviates T2DM-caused hyposalivation. A T2DM rat model was induced to evaluate the efficacy of BZYQF. The total saliva before and after acid stimulation was collected to determine the salivary flow rate and salivary alpha-amylase (sAA) activity. The parotid (PG) and submandibular glands (SMG) of experimental rats were removed to perform histopathology observation, biochemical indicator determination, and expression detection of signaling molecules in the salivary secretion pathway. **Results**: The present study screened out 1014 potential targets of BZYQF regarding the treatment of T2DM. These targets were mainly involved in the formation of the receptor complex, exercising the neurotransmitter receptor activity and regulating secretion. They were significantly enriched in the salivary secretion pathway of β1-AR/PKA/AMY1 and CHRM3/IP3R/AQP5. Furthermore, in BZYQF, nine validated compounds were able to dock into the active site of β1-AR, and three validated compounds were able to dock into the active site of CHRM3. Animal experiments confirmed that BZYQF significantly reduces fasting blood glucose, total cholesterol and triglyceride levels; enhances insulin level and HOMA-IS (*p* < 0.05); and increases salivary flow rate (Basal: increase from 21.04 ± 14.31 to 42.65 ± 8.84 μL/min, effect size of Cohen’s d = 6.80, *p* = 0.0078; Stimulated: increase from 36.88 ± 17.48 to 72.63 ± 17.67 μL/min, effect size of Cohen’s d = 7.61, *p* = 0.0025) and sAA activity (Basal: increase from 0.68 ± 0.32 to 2.17 ± 0.77 U/mL, effect size of Cohen’s d = 9.49, *p* = 0.0027; Stimulated: increase from 1.15 ± 0.77 to 4.80 ± 1.26 U/mL, effect size of Cohen’s d = 13.10, *p* = 0.0001) in basal and stimulated saliva in T2DM rats. Further mechanistic studies revealed that BZYQF reduces glucose and lipid accumulation, enhances acetylcholine content, improves pathological lesions and inflammation, and significantly increases the expression of salivary secretion pathway signaling molecules, including PKA, IP3R, β1-AR, AQP5, CHRM3, and AMY1 in the PG and SMG of T2DM rats (*p* < 0.05). **Conclusions**: The present study demonstrated that BZYQF is able to alleviate T2DM-caused hyposalivation by improving glucose metabolism and activating the salivary secretion pathway in the PG and SMG of T2DM rats. This study might provide a novel rationale and treatment strategy for BZYQF in diabetes-induced hyposalivation in a clinical setting.

## 1. Introduction

Type 2 diabetes mellitus (T2DM) is a chronic metabolic disorder characterized by hyperglycemia. The primary clinical symptoms include polydipsia, polyphagia, polyuria and weight loss. Polydipsia is an external manifestation of saliva secretion disorder in T2DM patients and is also a contributing factor to polyuria and polyphagia. Diabetes-caused hyposalivation not only alters salivary flow and its components but also causes a series of oral problems such as dysgeusia and oral ulcers and, in severe cases, can also lead to xerostomia [1,2]. A clinical report showed that the prevalence of xerostomia in diabetes patients was more than 50% [3]. It can be seen that improving saliva secretory function is of great importance in T2DM patients for maintaining oral health and blood glucose control.

Saliva consists of more than 99% water, along with electrolytes, and less than 1% protein components, including mucins, digestive enzymes such as amylase, and immunoglobulins. Saliva is mainly secreted by three major salivary glands, including the parotid gland (PG), the submandibular gland (SMG), and the sublingual gland (SLG) [2,4]. Salivary secretion is mainly regulated by both parasympathetic and sympathetic nerves. The parasympathetic nerves facilitate the secretion of fluid saliva including electrolytes, water, and a small amount of salivary proteins via acetylcholine, whereas the sympathetic nerves govern the secretion of protein saliva such as salivary alpha-amylase (sAA) via norepinephrine [5]. Moreover, the three major salivary glands respond differently to β-adrenergic stimuli. Under resting conditions, 65% of saliva (basal saliva) is secreted by the SMG. Under stimulated conditions, 50% of saliva (stimulated saliva) is produced by the PG [2,6]. However, the contribution of the SLG is minor to total saliva secretion. Additionally, salivary secretion is influenced by other factors such as age, gender, diet, medication, and disease [2,7]. Our previous studies found that hyposalivation in T2DM rats was closely associated with oxidative stress, inflammation and low expression of salivary secretion pathway signaling molecules in the PG and SMG [8]. Chae et al. also revealed that oxidative stress and its related endoplasmic reticulum stress response were closely related with T2DM-induced xerostomia [9,10]. In addition, a study showed that the disruption of tight junctions contributed to hyposalivation of salivary glands in T2DM mice [11].

At present, the drugs used to treat T2DM are mainly chemical drugs. These drugs have significant effects in managing blood glucose levels, but their effects on improving saliva secretion in T2DM patients are very limited [3]. Furthermore, they may cause side effects such as hypoglycemia, hepatorenal toxicity, and gastrointestinal discomfort [12]. The sialogogue drugs pilocarpine and cevimeline are often used to manage xerostomia. Both medications require some residual salivary gland function to be effective, as they work on muscarinic receptors and stimulate the patient’s glands to secrete saliva. Moreover, their most prevalent adverse effects increase sweating [13]. Despite the existing various new experimental approaches such as gene therapy, tissue engineering and cell-based therapy in regenerative therapy for damaged salivary glands, there are still many problems and challenges from bench to bedside [14]. Some clinical observations have revealed that mouthwashes and sprays made of traditional Chinese herbs can improve the xerostomia of diabetes patients [15,16]. This provides a new direction for the treatment of diabetes-caused hyposalivation. In traditional Chinese medicine (TCM) theory, diabetes belongs to the category of “XiaoKe disease” and its main pathogeny is spleen and stomach deficiency. As a TCM term, spleen and stomach deficiency refers to the impairment of the patient’s spleen and stomach functions. Moreover, TCM theory considers the spleen to be responsible for transportation and transformation of nutrients and saliva secretion. Patients with spleen and stomach deficiency demonstrate metabolic disorder and abnormal saliva secretion [17,18]. BuZhong Yiqi Formula (BZYQF) has been used in China for over 700 years. It is a classic formula for treating spleen and stomach deficiency and shows remarkable efficacy in improving digestive and immune functions [19,20]. Modern pharmacological research has revealed that BZYQF improves islet function and reduces hyperglycemia in T2DM patients [21,22]. However, there are few reports on BZYQF alleviating T2DM-caused hyposalivation.

This study first screened the core targets and key KEGG pathways involved in BZYQF’s improvement in saliva secretion in T2DM rats through network pharmacology methods. Then, we evaluated the ameliorative effect of BZYQF on blood glucose and saliva secretion in T2DM model rats through observing their salivary and biochemical indicators. Finally, we explored the molecular mechanism of BZYQF alleviating T2DM-caused hyposalivation by analyzing the histopathology and biochemical indicators, as well as gene expression of inflammatory factors and salivary secretion pathway signaling molecules in the PG and SMG of rats. The results of this study provide a theoretical basis for the use of BZYQF to treat T2DM and T2DM-caused hyposalivation.

## 2. Results

### 2.1. Network Pharmacology Analysis of BZYQF

**Figure 1:**
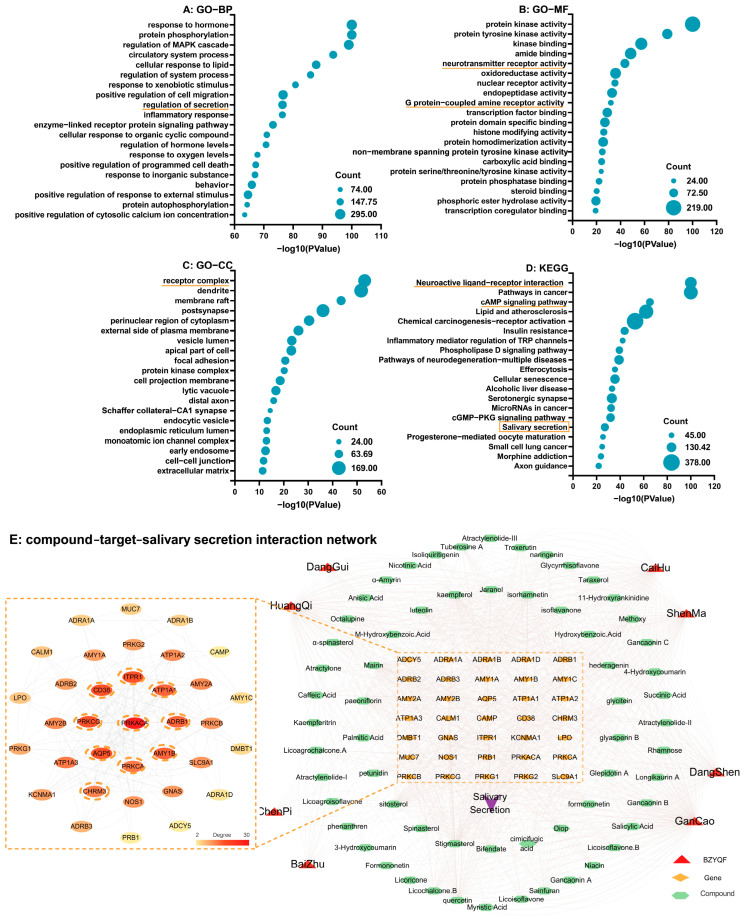
The enriched GO terms and KEGG pathways by the common targets of drug and disease and compound-target-salivary secretion pathway interaction network. The orange underline indicates the enriched GO-BP, MF, CC, and KEGG related to saliva secretion. The orange squares indicates the top 10 genes with the highest number of nodes in the compound-target-salivary secretion interaction network.

A total of 193 eligible compounds of BZYQF were screened using DL  ≥  0.18 and OB  ≥  30% or high absorption as filtering conditions, and they targeted 1109 targets. A total of 17,995 T2DM disease targets were extracted from the GeneCards database. A total of 1014 common targets were screened by matching 1109 drug targets and 17,995 disease targets. These 1014 common targets were potential targets of BZYQF for the treatment of T2DM. These potential targets were imported into Metascape for GO and KEGG enrichment analysis. The top 20 GO clusters and KEGG clusters are shown in Figure 1. These GO biological processes (BPs) were mainly involved in the response to substances or stimuli and the regulation of processes or substance, such as the regulation of secretion and inflammatory response (Figure 1A). These GO molecular functions (MFs) were mainly involved in enzyme or receptor activities, especially activities of kinase and neurotransmitter receptors and binding of enzymes or substances (Figure 1B). The receptor complex was the most significantly enriched cluster in GO cellular components (CC, Figure 1C). Among the KEGG pathways, the insulin resistance pathway was clearly enriched, and the neuroactive ligand-receptor interaction pathway was the most significantly enriched pathway. Furthermore, the cAMP signaling pathway and salivary secretion pathway were directly involved in saliva secretion (Figure 1D). The results of GO and KEGG analysis revealed that BZYQF alleviated T2DM via the insulin resistance pathway and ameliorated saliva secretion via the neurotransmitter-mediated salivary secretion pathway. As shown in Figure 1E, there were 35 genes enriched in the salivary secretion pathway, and these genes included key signaling molecules mediated by sympathetic and parasympathetic nerve in the salivary secretion pathway. The top 10 genes with the highest number of nodes in the compound-target-salivary secretion interaction network, which indicates that they were targeted by more active compounds, were PRKACA (PKA), AQP5, PRKCG, CD38, ITPR1 (IP3R), ATP1A1, ADRB1 (β1-AR), AMY1B (AMY1, coding gene of sAA), PRKCA, and CHRM3. These results suggested that BZYQF ameliorated hyposalivation in T2DM individuals mainly via the β1-AR/PKA/AMY1 pathway mediating salivary protein secretion and the CHRM3/IP3R/AQP5 pathway mediating salivary liquid secretion.

### 2.2. Molecular Docking of Validated Compounds in BZYQF

**Figure 2:**
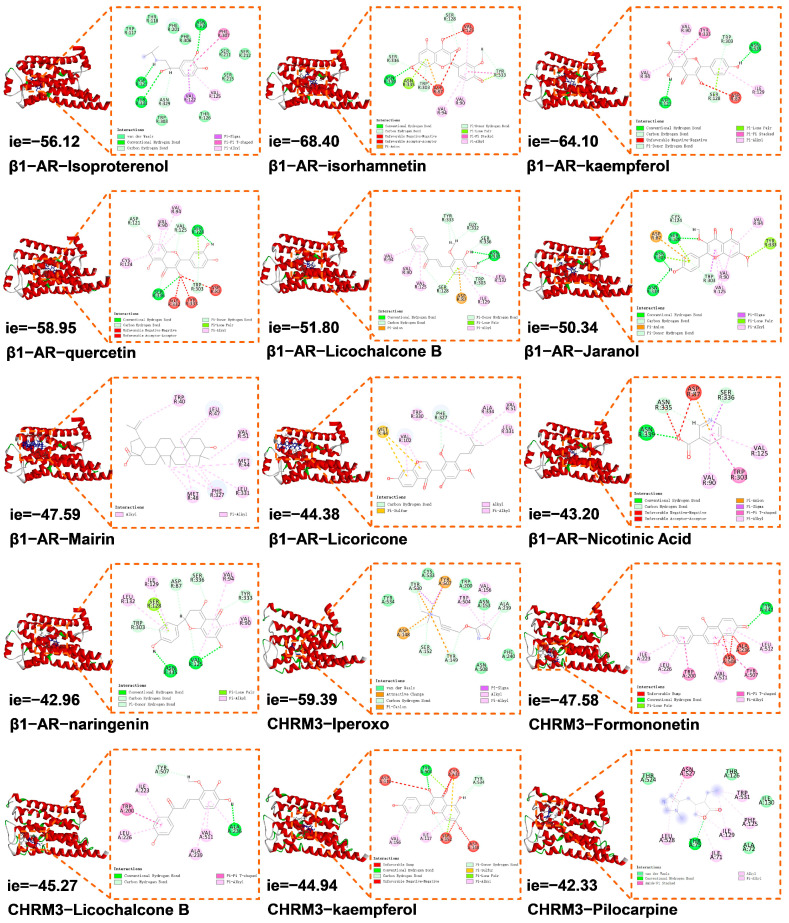
The docked 3D and 2D diagrams based on predicted optimal binding mode of validated compounds in BZYQF to β1-AR and CHRM3. They indicate CDOCKER interaction energy with a unit of Kcal/mol. Isoproterenol and iperoxo are agonists of β1-AR and CHRM3, respectively. Pilocarpine is a sialogogue drug working on muscarinic receptors.

Among the 16 compounds targeting β1-AR, 9 compounds were validated by widely targeted metabolomics data of BZYQF, including Jaranol, Mairin, quercetin, Licoricone, isorhamnetin, kaempferol, Licochalcone B, naringenin, and Nicotinic acid. Among the 14 compounds targeting CHRM3, 7 compounds were validated by widely targeted metabolomics data of BZYQF, including Atractylenolide I, Atractylenolide II, Formononetin, petunidin, kaempferol, Licochalcone B, and Mairin. As shown in Table 1, these validated compounds were flavonoids and terpenoids, mainly derived from GanCao, HuangQi and CaiHu. The molecular docking results showed that among these validated compounds, nine compounds were able to bind to β1-AR, and three compounds were able to bind to CHRM3, as their CDOCKER interaction energy was less than −40 Kcal/mol. As shown in Figure 2, compared with the agonist isoproterenol, the interaction energy of isorhamnetin and kaempferol binding to β1-AR was lower, while the interaction energy of other compounds binding to β1-AR was higher. Compared with the agonist iperoxo, the interaction energy of Formononetin, Licochalcone B, and kaempferol binding to CHRM3 was higher. However, their interaction energy was lower than that of the sialogogue drug pilocarpine binding to CHRM3. In addition, the interaction energy of kaempferol and Licochalcone B binding to β1-AR was lower than those binding to CHRM3. These results indicate that these validated compounds can bind effectively to the two targets and that they were more strongly bound to β1-AR than CHRM3.

As shown in Figure 2, the cryo-electron microscopy structure of isoproterenol -bound β1-AR (7JJO) revealed that isoproterenol was surrounded by 16 amino acid residues, including Trp 303, Val 125, Tyr 333, Asp 121, and so on, and they formed six interactions, including van der Waals, hydrogen and carbon hydrogen bonds, and Pi-sigma/Pi/alkyl interactions. The cryo-electron microscopy structure of iperoxo-bound CHRM3 (8EA0) revealed that iperoxo was surrounded by 14 amino acid residues, including Trp 200, Tyr 507, Ala 239, Val 156, and so on, and they formed seven interactions, including van der Waals, carbon hydrogen bond, attractive charge, Pi-cation/sigma/alkyl, and alkyl interactions. Compared with agonists (isoproterenol and iperoxo), these validated compounds of BZYQF docked into the same binding sites of β1-AR/CHRM3 and, docked into different binding sites of β1-AR and CHRM3, exhibiting active behavior. When bound to β1-AR, except for Mairin and Licoricone, the seven validated compounds were mainly surrounded by 12 amino acid residues of Trp 303, Val 125, Tyr 333, Asp 121, Val 90, Asp 87, Ser 128, Val 94, Asn 335, Ser 336, Asn 339, and Ile 129. Among these amino acid residues, the first four amino acids were binding sites of β1-AR with the agonist isoproterenol, and the remaining eight amino acids were binding sites of β1-AR with seven identified compounds. Mairin and Licoricone were bound to β1-AR mainly by four amino acid residues of Val 51, Met 44, Leu 331, and Phe 327, which were different from the binding sites of β1-AR with isoproterenol and the other seven validated compounds. In these cases, six main interactions of Pi-anion, Pi-alkyl, hydrogen, unfavorable negative-negative, Pi-lone pair, and carbon hydrogen bonds were formed between nine validated compounds and β1-AR. When bound to CHRM3, the three validated compounds were mainly surrounded by 14 amino acid residues of Trp 200, Tyr 507, Ala 239, Val 156, Trp 504, Cys 533, Tyr 534, Asp 148, Ser 152, Asn 508, Ala 236, Val 511, Leu 226, and Ile 223. Among these amino acid residues, the first 10 amino acids were binding sites of CHRM3 with the agonist iperoxo, and the remaining four amino acids were binding sites of CHRM3 with three identified compounds. In these cases, four main interactions of T-shaped Pi-Pi, Pi-alkyl, hydrogen, and carbon hydrogen bonds were formed between three validated compounds and CHRM3. In addition, when bound to CHRM3, the sialogogue drug pilocarpine was surrounded by 11 amino acid residues of Trp 531, Ala 72, Thr 26, Ile 130, and so on. These binding sites were different from the binding sites of the iperoxo and the three identified compounds with CHRM3. All these results indicated that on the one hand, similar to agonists, these compounds can dock to the active sites of β1-AR/CHRM3, exhibiting active behavior. On the other hand, unlike agonists, they are also able to dock to other unique binding sites of β1-AR/CHRM3, indicating a characteristic binding mode of β1-AR/CHRM3 with flavonoids.

### 2.3. BZYQF Alleviates T2DM

**Figure 3:**
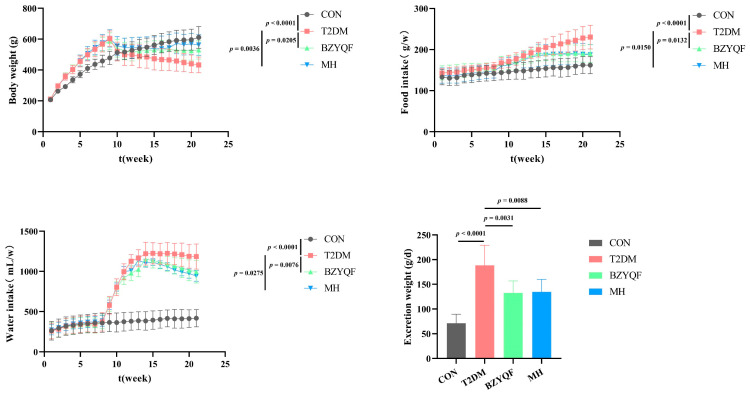
Daily indicators of experimental rats. CON group: N = 8; T2DM group: N = 8; BZYQF group, N = 8; MH group, N = 6.

At the initial stage of the experiment, no significant differences in body weight or intake of food and water were found among the experimental rats. After STZ injection for 2 weeks (10th week), disease model rats showed a significant decrease in body weight and a significant increase in water and food intake compared with CON rats. Furthermore, the padding in disease model rats was wetter than that in CON rats. The fasting blood glucose values of disease model rats were all >11.1 mM at three different times in 2 weeks, and disease model rats had lower fasting blood insulin (6.02 ± 1.25 vs. 14.09 ± 1.93 μU/mL) and HOMA-IS (6.85 ± 1.53 vs. 118.23 ± 40.31) than CON rats. After a T2DM rat model was successfully induced, rats in the BZYQF group were interfered with. After drug intervention for 11 weeks (21st week), BZYQF and MH significantly increased body weight and reduced food intake, water intake, and excretion weight in T2DM rats (Figure 3). Serum biochemical index determination (Table 2) indicated that BZYQF and MH significantly reduced glucose (*p*_BZYQF_ = 0.0012 and *p*_MH_ = 0.0012) and TG levels (*p*_BZYQF_ = 0.0288 and *p* _MH_ = 0.0195) and increased Ach level (*p* _BZYQF_ < 0.0001 and *p* _MH_ < 0.0001) in T2DM rats. In addition, BZYQF significantly increased FINS content (*p* = 0.0024) and HOMA-IS (*p* = 0.0019) and reduced TC level (*p* = 0.0348) in T2DM rats, while MH had no effect on the TC level (*p* > 0.05) in T2DM rats. These results revealed that BZYQF can alleviate T2DM symptoms and improve glucose and lipid metabolism in T2DM rats.

### 2.4. BZYQF Ameliorated Saliva Secretion and Responsiveness to Citric Acid Stimulation in T2DM Rats

**Figure 4:**
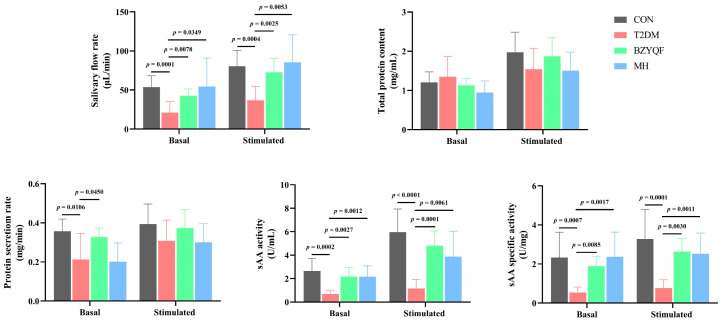
Basal and stimulated salivary parameters in experimental rats. CON group: N = 8; T2DM group: N = 8; BZYQF group, N = 8; MH group, N = 6.

To evaluate the improvement effect of BZYQF on salivary secretion in T2DM rats, we collected and measured the salivary indicators before and after citric acid stimulation. As presented in Figure 4, the salivary flow rate (Basal effect size of Cohen’s d = 8.46, *p* = 0.0001; Stimulated effect size of Cohen’s d = 8.57, *p* = 0.0004), sAA activity, (Basal effect size of Cohen’s d = 9.37, *p* = 0.0002; Stimulated effect size of Cohen’s d = 11.99, *p* < 0.0001) and sAA-specific activity (Basal effect size of Cohen’s d = 7.14, *p* = 0.0007; Stimulated effect size of Cohen’s d = 8.46, *p* = 0.0001) in basal and stimulated saliva of T2DM rats were significantly lower than those of CON rats. Moreover, the protein secretion rate in basal (Effect size of Cohen’s d = 5.17, *p* = 0.0106) but not stimulated saliva (*p* > 0.05) of T2DM rats was significantly lower than that of CON rats. After drug intervention, BZYQF and MH significantly increased the salivary flow rate (BZYQF: Basal effect size of Cohen’s d = 6.80, *p* = 0.0078; Stimulated effect size of Cohen’s d = 7.61, *p* = 0.0025. MH: Basal effect size of Cohen’s d = 4.45, *p* = 0.0349; Stimulated effect size of Cohen’s d = 6.35, *p* = 0.0053), sAA activity (BZYQF: Basal effect size of Cohen’s d = 9.49, *p* = 0.0027; Stimulated effect size of Cohen’s d = 13.10, *p* = 0.0001. MH: Basal effect size of Cohen’s d = 7.85, *p* = 0.0012; Stimulated effect size of Cohen’s d = 6.21, *p* = 0.0061), and sAA-specific activity (BZYQF: Basal effect size of Cohen’s d = 12.95, *p* = 0.0085; Stimulated effect size of Cohen’s d = 12.47, *p* = 0.0030. MH: Basal effect size of Cohen’s d = 7.54, *p* = 0.0017; Stimulated effect size of Cohen’s d = 7.98, *p* = 0.0011) in basal and stimulated saliva of T2DM rats. Furthermore, BZYQF notably enhanced the protein secretion rate (Effect size of Cohen’s d = 4.29, *p* = 0.0450) in basal saliva of T2DM rats and had a tendency to increase the protein secretion rate (Effect size of Cohen’s d = 2.46, *p* = 0.0658) in stimulated saliva. Similar results had not been found in MH (*p* > 0.05). Additionally, there was no difference observed in the total protein content in basal and stimulated saliva among the rats of the four groups (*p* > 0.05).

The delta value of the salivary parameters reflected the responsiveness of experimental rats to citric acid stimulation. As shown in Table 3, after citric acid stimulation, the salivary flow rate (Effect size of Cohen’s d = 5.65, *p* = 0.0092) and sAA activity (Effect size of Cohen’s d = 7.82, *p* = 0.0009) clearly increased, while the sAA-specific activity, the total protein content and the protein secretion rate changed less in CON rats (*p* > 0.05). In addition, the delta values of salivary flow rate (Effect size of Cohen’s d = 4.23, *p* = 0.0402), total protein content (Effect size of Cohen’s d = 4.12, *p* = 0.0449), sAA activity (Effect size of Cohen’s d = 11.02, *p* < 0.0001), and sAA-specific activity (Effect size of Cohen’s d = 4.24, *p* = 0.0398) in T2DM rats were significantly lower than in CON rats. After drug intervention, BZYQF significantly increased the delta values of salivary flow rate (Effect size of Cohen’s d = 4.82, *p* = 0.0221), total protein content (Effect size of Cohen’s d = 4.14, *p* = 0.0438), sAA activity (Effect size of Cohen’s d = 10.66, *p* < 0.0001), and sAA-specific activity (Effect size of Cohen’s d = 4.32, *p* = 0.0368) in T2DM rats. MH only increased the delta values of salivary flow rate (Effect size of Cohen’s d = 5.31, *p* = 0.0149) and sAA activity (Effect size of Cohen’s d = 4.65, *p* = 0.0286) but not the delta values of total protein content (*p* > 0.05) and sAA-specific activity (*p* > 0.05) in T2DM rats. Furthermore, the delta values of total protein content, sAA activity, and sAA-specific activity in the rats of the BZYQF group were significantly higher than those in the rats of the MH group (*p* < 0.05). In addition, there were no differences in the delta values of the protein secretion rate among the four groups (*p* > 0.05). These results revealed that BZYQF alleviates T2DM-caused hyposalivation, manifested by ameliorating basal and stimulated saliva secretion, as well as responsiveness to citric acid stimulation in T2DM rats.

### 2.5. BZYQF Improved the Levels of Biochemical Indicators and sAA Activity in the PG and SMG of T2DM Rats

To investigate the histological reasons for BZYQF improving saliva secretion in T2DM rats, we measured the biochemical indicators of the PG and SMG in the experimental rats. As shown in Table 4, the glucose content (*p* _PG_ < 0.0001 and *p* _SMG_ = 0.0010) in the PG and SMG of T2DM rats was significantly higher than that in CON rats, while the sAA activity (*p* _PG_ = 0.0008 and *p* _SMG_ = 0.0001) and Ach content (*p* _PG_ < 0.0001 and *p* _SMG_ = 0.0004) in the PG and SMG of T2DM rats were significantly lower than those in CON rats. After drug intervention, BZYQF significantly reduced glucose content (*p* _PG_ = 0.0261 and *p* _SMG_ = 0.0012) and enhanced sAA activity (*p* _PG_ = 0.0025 and *p* _SMG_ = 0.0012) and Ach content (*p* _PG_ = 0.0126 and *p* _SMG_ = 0.0041) in the PG and SMG of T2DM rats. In addition, in the PG, T2DM rats had notably higher contents of TC (*p* = 0.0011) and TG (*p* = 0.0003) than CON rats. However, BZYQF significantly reduced TG content (*p* = 0.0185) and had a tendency to decrease TC content (*p* = 0.0698) in the PG of T2DM rats. In the SMG, no significant differences in the contents of TC and TG were observed among the rats of three groups (*p* > 0.05). There was also no significant difference in the total protein content of PG and SMG among the rats of three groups (*p* > 0.05). Interestingly, the contents of glucose, TC, and TG in the PG of all experimental rats were significantly higher than those in the SMG (*p* < 0.05). Conversely, the sAA activity, Ach and total protein contents in the PG of all experimental rats were lower than those in the SMG (*p* < 0.05). These results revealed that BZYQF alleviated the accumulation of glucose and lipid and increased sAA activity and Ach content in the PG and SMG of T2DM rats. At the same time, these results also suggested that there was a functional difference in the PG and SMG of rats, and T2DM caused greater damage to the PG than to the SMG.

### 2.6. BZYQF Improved the Morphological Structure and sAA Content in the PG and SMG of T2DM Rats

**Figure 5:**
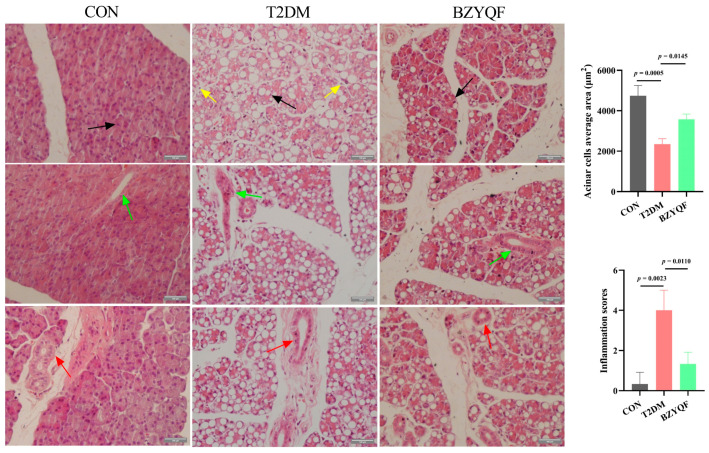
HE staining of PG tissue in experimental rats (400×). Black arrows indicate acinar cells, yellow arrows indicate inflammatory cells, green arrows indicate secretory ducts, and red arrows indicate interlobular ducts. CON group: N = 3; T2DM group: N = 3; BZYQF group, N = 3.

In order to further explore the histological reasons for BZYQF improving saliva secretion in T2DM rats, we observed the histopathological changes in the PG and SMG of experimental rats. The HE staining in the PG is shown in Figure 5. In the PG of CON rats, the acinar cells had a normal structure, neat arrangement, and regular shape, with a large number of secretory granules. The secretory ducts and interlobular ducts had a regular morphology and distinctly visible lumens surrounded by evenly distributed cells. In the PG of T2DM rats, the acinar cells had a loose structure, disorderly arrangement and irregular shape, with reduced secretory granules. Moreover, there was extensive vacuolization in the acinar cells, accompanied by infiltration of inflammatory cells. Acinar cell area was reduced, and inflammatory infiltration scores were increased. Additionally, in the PG of T2DM rats, the secretory ducts had collapsed and atrophied, and interlobular ducts showed varying levels of deformation and dilated lumens. The cells surrounding the ducts had an irregular arrangement. After intervention with BZYQF, the acinar cells in T2DM rats had a significant improvement in the structure and arrangement of their cells, manifested by a decrease in vacuolization and inflammatory infiltration, an increase in secretory granules and acinar cell area, and clear visibility of the lumens of secretory ducts and interlobular ducts.

**Figure 6:**
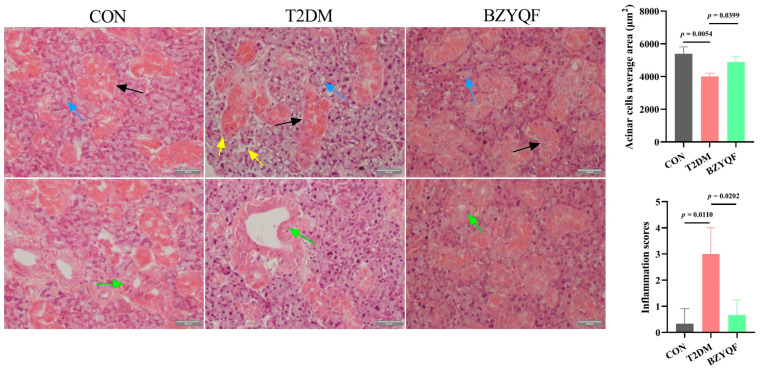
HE staining of SMG tissue in experimental rats (400×). Blue arrows indicate serous acinar cells, black arrows indicate mucinous acinar cells, yellow arrows indicate inflammatory cells, and green arrows indicate secretory ducts. CON group: N = 3; T2DM group: N = 3; BZYQF group, N = 3.

HE staining in the SMG is shown in Figure 6. In the SMG of CON rats, the serous and mucinous acinar cells had a normal structure, neat arrangement, and regular shape. The secretory ducts had clear and visible lumen surrounded by neatly arranged cells. In the PG of T2DM rats, the acinar cells had a loose structure, accompanied by the infiltration of inflammatory cells and vacuolization of some serous acinar cells. Acinar cell area was reduced, and inflammatory infiltration scores were increased. Additionally, their secretory duct lumen was enlarged and deformed. After BZYQF intervention, the acinar cells and secretory ducts of T2DM rats were significantly alleviated in terms of their morphological structure, and inflammatory infiltration of T2DM rats was clearly reduced.

HE staining of the PG and SMG in experimental rats indicated that the two salivary glands had similar glandular structure, but T2DM caused greater damage to the PG than to the SMG, manifested by incomplete morphology, higher vacuolization, and inflammatory infiltration in the acinar cells of the PG.

**Figure 7:**
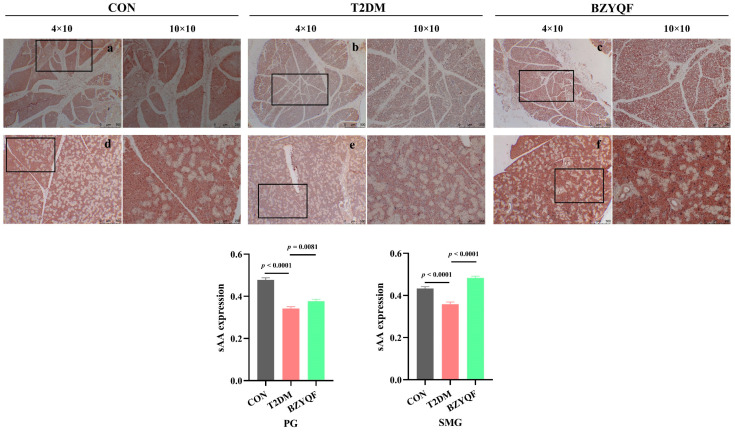
IHC staining for sAA in the PG and SMG tissue of experimental rats. (**a**–**c**) PG; (**d**–**f**) SMG. Magnification is 40× and 100×. CON group: N = 3; T2DM group: N = 3; BZYQF group, N = 3.

Immunohistochemical (IHC) staining for sAA in the PG and SMG is shown in Figure 7. sAA staining was observed in the serous acinar cells of the PG and SMG. The PG and SMG of CON rats had stronger sAA staining, appearing dark brown, while the sAA staining in the PG and SMG of T2DM rats was significantly reduced, appearing light brown. After drug intervention, BZYQF markedly enhanced the sAA staining intensity in the PG and SMG of T2DM rats. These results revealed that BZYQF was able to increase the sAA content in the PG and SMG of T2DM rats.

### 2.7. BZYQF Upregulated mRNA Expression of Inflammatory Factors and Salivary Secretion Pathway Signaling Molecules in the PG and SMG of T2DM Rats

**Figure 8:**
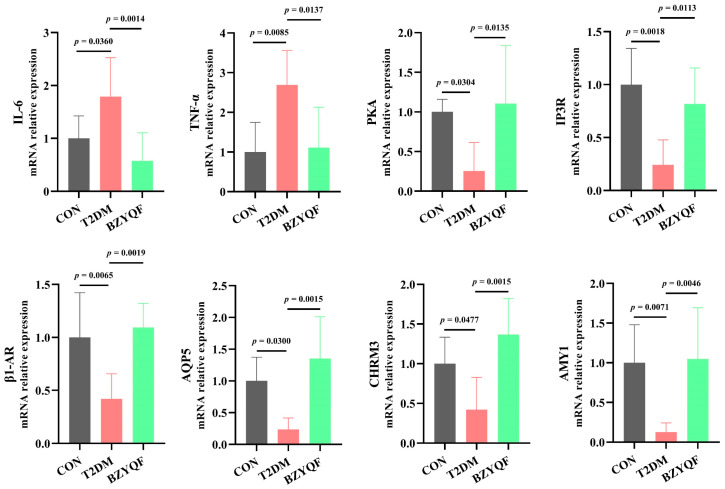
The mRNA expression of inflammatory factors and salivary secretion pathway signaling molecules in the PG of experimental rats. CON group: N = 6–8; T2DM group: N = 6–8; BZYQF group, N = 6–8.

**Figure 9:**
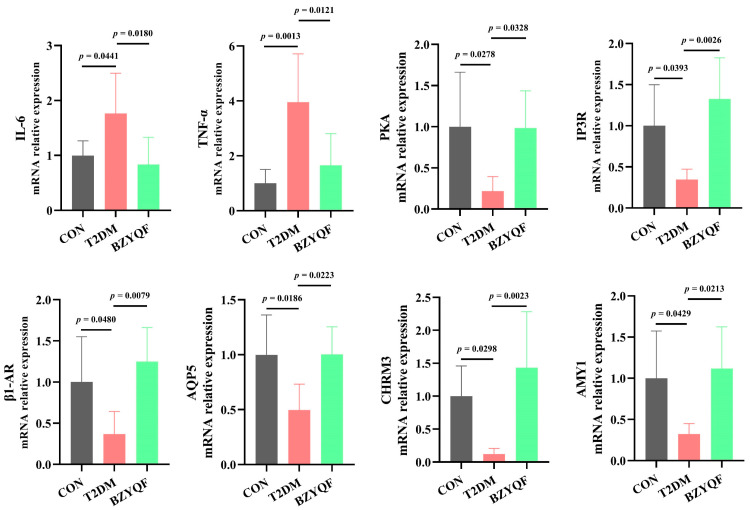
The mRNA expression of inflammatory factors and salivary secretion pathway signaling molecules in the SMG of experimental rats. CON group: N = 6–8; T2DM group: N = 6–8; BZYQF group, N = 6–8.

To investigate the molecular mechanisms of BZYQF in alleviating hyposalivation in T2DM rats, we detected the mRNA expression of signaling molecules of the salivary secretion pathway in the PG and SMG of the experimental rats. As presented in Figure 8 and Figure 9, the mRNA expression of salivary secretion pathway signaling molecules, including PKA, IP3R, β1-AR, AQP5, CHRM3, and AMY1, was significantly lower in the PG and SMG of T2DM rats than in those of CON rats, and the mRNA expression of inflammatory factors, such as IL-6 and TNF-α, was significantly higher in the PG and SMG of T2DM rats than in those of CON rats. After drug intervention, BZYQF markedly enhanced the mRNA expression of PKA, IP3R, β1-AR, AQP5, CHRM3, and AMY1 and reduced the mRNA expression of IL-6 and TNF-α in the PG and SMG of T2DM rats.

### 2.8. BZYQF Upregulated Protein Expression of β1-AR, sAA, and CHRM3 of Salivary Secretion Pathway in the PG and SMG of T2DM Rats

**Figure 10:**
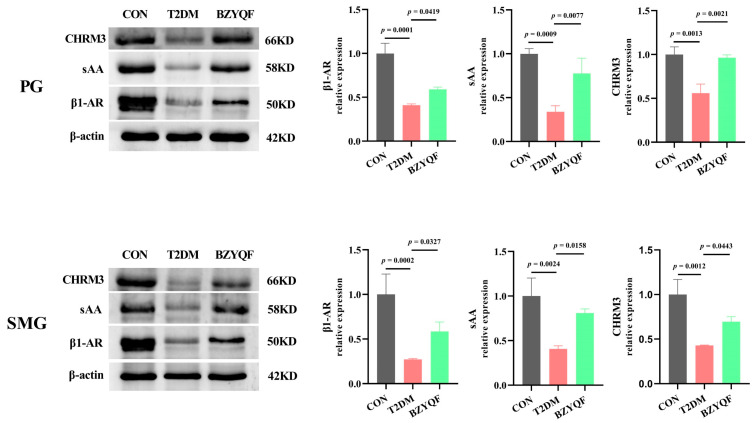
The protein expression of β1-AR, sAA and CHRM3 in the PG and SMG of experimental rats. CON group: N = 3; T2DM group: N = 3; BZYQF group, N = 3.

To further investigate the molecular mechanisms of BZYQF alleviating hyposalivation in T2DM rats, we detected the protein expression of β1-AR, sAA and CHRM3 of the salivary secretion pathway in the PG and SMG of the experimental rats. As presented in Figure 10, the protein expression of β1-AR, sAA, and CHRM3 in the PG and SMG of T2DM rats was significantly lower than those of CON rats. After drug intervention, BZYQF significantly upregulated the protein expression of β1-AR, sAA, and CHRM3 in the PG and SMG of T2DM rats. The detection results of protein expression were consistent with those of mRNA expression, which revealed that BZYQF can enhance the expression of salivary secretion pathway signaling molecules in the PG and SMG of T2DM rats.

## 3. Discussion

The present study first predicted that BZYQF could alleviate hyposalivation through targeting signaling molecules in the salivary secretion pathway using network pharmacology analysis and molecular docking. Subsequently, animal experiments revealed that BZYQF was able to alleviate symptoms of T2DM in rats and improve the secretion of saliva before and after acid stimulation in T2DM rats. Mechanism studies confirmed that BZYQF ameliorated histopathological changes and gene expression levels of salivary secretion pathway signaling molecules in the PG and SMG of T2DM rats. This study provides a theoretical basis for the use of BZYQF in the treatment of T2DM and T2DM-caused hyposalivation, which has practical significance for the treatment of T2DM.

Chinese herbal formulas are a combination of multiple Chinese herbs according to the principle of compatibility in TCM, and are used to treat relatively specific symptoms of diseases. These formulas demonstrate multicomponent, multitarget, multipathway, and synergistic effects. Network pharmacology uses the theory of systems biology to construct complex drug-target-disease networks and analyze the interactions among drug, target, and disease. Molecular docking is a drug design method which, based on structure, can predict the binding ability and action mode between a drug and a target. The combination of network pharmacology and molecular docking can comprehensively and systematically reveal the pharmacological substance basis and molecular mechanism of Chinese herbal formulas, promoting in-depth research and clinical application of Chinese herbs. This combination has been widely used in various fields researching traditional Chinese herbs [23,24]. This study screened out 1014 potential targets of BZYQF for the treatment of T2DM via network pharmacology analysis. These potential targets were mainly involved in the formation of the receptor complex, exercised the activities of kinase and the neurotransmitter receptor, and participated in regulation of the secretion and inflammatory response. KEGG analysis indicated that these potential targets were significantly enriched in the pathways of insulin resistance and salivary secretion. The interaction network of the compound-target-salivary secretion pathway revealed that the top 10 genes of PKA, AQP5, PRKCG, CD38, IP3R, ATP1A1, β1-AR, AMY1, PRKCA, and CHRM3 participated in regulating saliva secretion via the β1-AR/PKA/AMY1 pathway and CHRM3/IP3R/AQP5 pathway. Molecular docking confirmed that the nine validated compounds of Jaranol, Mairin, quercetin, Licoricone, isorhamnetin, kaempferol, Licochalcone B, naringenin, and Nicotinic acid were able to dock into the active site of β1-AR, exhibiting activated binding behavior, and the three validated compounds of Formononetin, kaempferol, and Licochalcone B were able to, dock into the active site of CHRM3, exhibiting activated binding behavior. Furthermore, when bound to CHRM3, the three compounds of Formononetin, kaempferol, and Licochalcone B had different binding sites from the sialogogue drug pilocarpine, and they exhibited better affinity to CHRM3. These results suggested that BZYQF might alleviate T2DM-caused hyposalivation by regulating the salivary secretion pathway.

Salivary gland damage caused by T2DM is the main cause of hyposalivation and subsequent polydipsia in T2DM patients. Polydipsia in T2DM patients not only can cause taste disorder and overeating but can also cause abnormal water and salt metabolism, resulting in polyuria and weight loss. Therefore, it is of great significance to alleviate the salivary gland damage caused by T2DM. At present, the drugs used to treat T2DM have a significant effect in controlling blood glucose but have less effect on saliva secretion. On the contrary, some hypoglycemic drugs and some drugs used to treat other comorbidities, such hypertension and neurological diseases, may worsen dry mouth and cause nausea, abdominal distension, retention of water and sodium, hypoglycemia, and other side effects [12]. At present, saliva substitutes and saliva stimulants (e.g., pilocarpine and cevimeline) are commonly used to improve saliva secretion. However, these treatments do not facilitate the repair and regeneration of damaged salivary gland tissue [25]. Consequently, it is of great importance to develop a drug that can simultaneously reduce blood glucose levels and enhance the function of salivary glands in order to improve blood glucose control and the quality of life of patients with T2DM. In recent years, clinical observations suggested that ginger-aloe vera mouthwash and dark plum-lemon spray had improving effects on the xerostomia of diabetes patients [15,16]. In addition, some studies found that *ixeris dentata* and *lactobacillus gasseri* extracts was able to increase salivary secretion in diabetes-induced xerostomia rats by regulating oxidative stress and its related endoplasmic reticulum stress response [9,10]. Kim et al. found that polydatin, a plant polyphenol, was able to alleviate diabetes-induced hyposalivation via anti-glycation activity in db/db mice [26]. These results suggest that herbal extracts or their active ingredients can improve diabetes-induced xerostomia or hyposalivation. BZYQF is a classic TCM formula for treating spleen and stomach deficiencies that cause digestive and metabolic disorders. Modern pharmacological research has revealed that BZYQF and the active constituents of single herbs from BZYQF, such as *Astragalus* polysaccharide, *Codonopsis pilosula* polysaccharidem and saikosaponin D, have a definite hypoglycemic effect in T2DM individuals [21,22,27,28,29,30,31]. However, the effect of BZYQF on saliva secretion in T2DM individuals has not yet been reported. Saliva is composed of two parts: liquid saliva and protein saliva. Liquid saliva mainly includes water and electrolytes, which are responsible for wetting the oral cavity, dissolving food, and promoting swallowing. Protein saliva mainly consists of amylase, mucin, lysozyme, and so on, which are responsible for digesting starch and maintaining oral health. Salivary flow rate represents the secreted volume of saliva per unit of time and is often used to evaluate the secretion of liquid saliva. The sAA is the main component of salivary proteins, and its activity is an important indicator for evaluating the secretion of protein saliva. Acid (especially citric acid) stimulation, as a means of stimulating saliva secretion, has been used to evaluate the malnutrition status and salivary gland function of individuals with metabolic disorders, including T2DM [8,32,33,34]. Clinical studies have found that salivary flow rate and sAA activity in basal saliva were significantly reduced in T2DM patients compared with healthy individuals and that the composition of the saliva of T2DM patients was also significantly altered [1,2,35]. Moreover, many studies have found that sAA activity was negatively correlated with the occurrence of metabolic diseases such as obesity and diabetes [36,37]. This study found that BZYQF can significantly alleviate the symptoms of T2DM rats; reduce the levels of glucose, TC, and TG in serum; and increase the levels of Ach and FINS. These results confirm that BZYQF has good hypoglycemic, lipid-lowering, and insulin secretion-promoting effects. Moreover, this study found that T2DM rats had impaired secretion of basal and stimulated saliva and a decreased response to acid stimulation compared with CON rats. However, BZYQF can significantly increase the reduced salivary flow rate, sAA activity, and sAA-specific activity in basal and stimulated saliva of T2DM rats and significantly enhance the reduced delta values of salivary flow rate, total protein content, sAA activity, and sAA-specific activity in T2DM rats. These results reveal that BZYQF can alleviate hyposalivation caused by T2DM. In addition, this study found that there was no difference in the total protein content of basal and stimulated saliva among the rats of the CON, T2DM, and BZYQF groups. This might be attributed to the simultaneous reduction in liquid saliva and protein saliva of T2DM rats. In the present study, the protein secretion rate in basal saliva of T2DM rats was significantly lower than that of CON rats and BZYQF rats. This might be related to the prolonged time of basal saliva collection caused by the hyposalivation in T2DM rats.

The salivary glands are an exocrine organ responsible for the production and secretion of saliva, mainly including the PG, SMG, and SLG, as well as the secondary salivary glands distributed in the oral cavity [4]. The PG is responsible for saliva secretion under stimulated conditions, while the SMG is responsible for saliva secretion under resting conditions. The two glands secrete about 85% of saliva and are the main glands responsible for saliva secretion [5]. Salivary glands are mainly composed of dendritic branching ducts and the terminal acinus. The acinus are divided into the serous acinus and mucinous acinus, which can secrete zymogen granules. The PG is a pure serous gland and consists of serous acinus. The SMG is a mixed gland consisting of serous acinus and mucinous acinus, and the serous acinus is predominant [38]. The ducts mainly include intercalary ducts, secretory ducts, interlobular ducts, and so on. Saliva produced by the acinus reaches the oral cavity via these ducts [39]. The ducts are involved in the bidirectional transport and reabsorption of electrolytes, producing saliva with low osmotic pressure and secreting small granules containing potassium ions and glycoproteins [40]. Our previous studies demonstrated that there were varying degrees of histopathological lesions in the PG and SMG of T2DM rats, and the PG of T2DM rats suffered more severe damage in morphological structure, oxidative stress, and inflammatory levels than the SMG [8]. Similar reports found that diabetes caused damage to all three salivary glands, but the PG was more damaged by diabetes [41,42]. Hassan et al. found that diabetes was able to cause glandular atrophy of the PG parenchyma, characterized by the loss of gland structure, degenerated acini, and dilatation of the duct system. These structural changes interfered with saliva production and secretion, leading to xerostomia [43]. Takahashi et al. confirmed that the decrease in salivary secretion from the SMG of T2DM mice was closely related to the increase in expression levels of TNF-α and IL-1β and number of lymphocyte infiltration points in their tissues [44]. This study found that acinar cells in the PG and SMG of T2DM rats exhibited significant vacuolization and ductal deformation accompanied by an infiltration of inflammatory cells and an increase in the levels of expression of TNF-α and IL-6 and that the PG of T2DM rats was more prominent. However, BZYQF can significantly improve these histopathological lesions and reduce the levels of expression of TNF-α and IL-6 in the PG and SMG of T2DM rats. In addition, the excessive accumulation of glucose and lipids in salivary glands may induce inflammation and oxidative stress. Our previous research found that an increase in inflammation and oxidative stress levels in the salivary glands of T2DM rats was associated with an increase in glucose and lipid content [8]. This study found that the glucose content was significantly increased in the PG and SMG of T2DM rats compared with those of CON rats. However, BZYQF can significantly reverse this indicator. Moreover, the levels of TC and TG in the PG of T2DM rats were significantly higher than those of CON rats, but similar results were not found in the SMG. BZYQF can only improve the TG content in the PG of T2DM rats. These results suggest that T2DM caused the accumulation of glucose and lipid in the salivary glands, especially the PG, damaging the function of the salivary glands, and that BZYQF could improve this process. Interestingly, the present study found that the levels of glucose, TC, and TG in the PG of experimental rats were higher, while the sAA activity and Ach content were lower than those in the SMG. This result suggest a difference in salivary secretion function between the PG and SMG and also reflect that T2DM might cause greater damage to the PG than to the SMG.

The saliva from the salivary glands is mainly regulated by the autonomic nervous system. The sympathetic nervous system regulates the secretion of protein saliva by activating the β-AR/Gs/AC/cAMP/PKA signaling pathway via norepinephrine (NA). The parasympathetic nervous system regulates the secretion of fluid saliva by activating the CHRM3/Gq/PLCβ/IP3R/Ca^2+^/AQP5 signaling pathway via Ach [45,46]. Diabetic neuropathy is the most frequent complication of T2DM and can affect up to 50% of patients with T2DM during their lifetime [47]. KOŁODZIEJ et al. [42] found that when stimulated by the muscarinic agonist pilocarpine, the salivary flow rate in rats with HFD caused insulin resistance to decrease by 45% compared with rats of the control group. The present study found that BZYQF can significantly increase the decreased Ach content in the serum, PG, and SMG of T2DM rats. This result suggests that BZYQF can improve saliva secretion in T2DM rats by increasing Ach content. AQP5 is the first aquaporin identified in salivary glands. Research has demonstrated that the saliva in mice with a knockout of the AQP5 gene decreased by more than 60% compared with wild mice and has reported alterations in the composition of their saliva [48]. Our previous research found that the PG and SMG of T2DM rats exhibited decreased expression of signaling molecules in the salivary secretion pathway [8]. The present study found that BZYQF can significantly increase the reduced expression of salivary secretion pathway signaling molecules in the PG and SMG of T2DM rats, including PKA, IP3R, β1-AR, AQP5, CHRM3, and AMY1. Our immunohistochemistry and Western blot results further confirmed the improvement effect of BZYQF on the protein expression of CHRM3 and β1-AR in the PG and SMG of T2DM rats. These results suggest that BZYQF can improve saliva secretion in T2DM rats by activating the salivary secretion pathway. In addition, this study found that the activity and/or content and/or gene expression of sAA in the saliva, PG, and SMG of T2DM rats were significantly lower than those of CON rats, indicating the impairment of sAA secretion and synthesis in T2DM rats. However, BZYQF reversed these indicators, indicating that BZYQF can simultaneously improve the secretion and synthesis of sAA.

Taken together, the findings of this study provide new possibilities for the use of BZYQF to treat diabetes-caused xerostomia or hyposalivation. BZYQF is different from general hypoglycemic drugs and sialogogue drugs. While reducing blood glucose level, BZQYF can also repair the morphological structure and function of salivary glands, increasing the secretion of saliva. Hence, BZYQF may treat T2DM-caused hyposalivation from the etiology, which has important clinical practical significance. In addition, as a classic formula, BZYQF has a history of hundreds of years of use in East Asia. Many Chinese herbs in BZYQF, such as *astragalus membranaceus*, *codonopsis pilosula*, *glycyrhiza uralensis*, *atractylodes macrocephala*, *citrus reticulata*, *zingiber offcinale*, and *ziziphus jujuba*, are homologous with medicine and food and have proven to be safe in clinical practice. Furthermore, BZYQF is mainly used to regulate digestive and metabolic disorders in the clinical practice of TCM, and it has a good regulatory effect on the gastrointestinal tract. At present, there are no relevant reports mentioning the side effects of BZYQF, including the gastrointestinal discomfort that often occurs with chemical drugs.

## 4. Materials and Methods

### 4.1. Preparation and Constituent Characterization of BZYQF

BZYQF was manufactured in pill form by JiuZhiTang Co., Ltd. (National Medical Approval No. Z43020143, batch number 202209087, Hunan, Changsha, China) in accordance with the drug standards of the Ministry of Health for Traditional Chinese Medicine Formulations (Volume 7; Standard Number: WS3-B-1347-93). The components and dosage of Chinese herbs in BZYQF are shown in Table 5. The chemical constituent characterization of BZYQF was performed using the widely targeted metabolomics techniques based on UPLC-MS/MS. The calycosin-7-O-β-D-glucoside, the flavonoid with the highest content in BZYQF, was quantitated by HPLC. The main constituent contents in BZYQF were determined using the colorimetric method. The widely targeted metabolomics identified a total of 1909 compounds in BZYQF. The compounds accounting for more than 5% were, in descending order, flavone, phenolic acids, amino acids and their derivatives, alkaloids, lipids, terpenoids, organic acids, lignans, and coumarins. Flavone accounted for about 25% and contained 467 secondary flavonoids, which mainly included flavone, xanthone alcohol, isoflavone, dihydroflavonoid, chalcone, and other flavonoids (as shown in Supplementary Materials). The content of calycosin-7-O-β-D-glucoside was 0.83 mg/g in each pill. The total carbohydrate content was 146.72 ± 2.59 mg/g. The reducing sugar content was 86.22 ± 1.11 mg/g. The polysaccharide content was 60.50 ± 3.12 mg/g. The total flavonoid content was 23.45 ± 4.39 mg/g. The total polyphenol content was 123.79 ± 2.68 mg/g. The total saponin content was 8.11 ± 3.05 mg/g. A detailed report was shown in our previous research [49].

### 4.2. Network Pharmacology Analysis

The chemical compounds of eight Chinese herbs in BZYQF—namely, HuangQi, DangShen, GanCao, BaiZhu, DangGui, ChenPi, ShengMa, and ChaiHu—were retrieved through the databases of TCMSP 2.3 (https://www.tcmsp-e.com; accessed on 5 May 2024) and ETCM 2.0 (http://www.tcmip.cn/ETCM2/front/#/; accessed on 5 May 2024). The active chemical compounds were screened according to the inclusion criteria of druglikeness (DL) ≥ 0.18 and oral bioavailability (OB) ≥ 30% in the TCMSP database, along with the discriminant criteria of DL ≥ 0.18 and high absorption in the SwissADME database (http://www.swissadme.ch/; accessed on 10 May 2024). The SMILES numbers of these active compounds were retrieved through the PubChem database (https://pubchem.ncbi.nlm.nih.gov/; accessed on 15 May 2024) and imported into SwissTargetPrediction (http://www.swisstargetprediction.ch; accessed on 15 May 2024) to predict their potential targets. Targets filtered with “Homo sapiens” and “Probability > 10” were downloaded. The human disease targets of T2DM were searched for in GeneCards 5.21 (https://www.genecards.org/; accessed on 15 May 2024). The common targets of BZYQF and T2DM were obtained using the online software jvenn (https://jvenn.toulouse.inra.fr; accessed on 15 May 2024). Finally, these common targets were subjected to Gene Ontology (GO) and Kyoto Encyclopedia of Genes and Genomes (KEGG) enrichment analysis using Metascape 3.5 (https://metascape.org; accessed on 10 June 2024). The compound-target-pathway interaction network was constructed using the Cytoscape 3.10.0 software, and the core targets of BZYQF against T2DM-caused hyposalivation were determined through the number of nodes between the active compounds and the targets in the interaction network.

### 4.3. Molecular Docking

The active compounds targeting β1-AR and CHRM3 were validated using widely targeted metabolomics data of BZYQF. Molecular docking was applied to generate possible binding site(s) between β1-AR/CHRM3 and these validated active compounds in BZYQF using Discovery Studio 2019. Considering the existing cryo-electron microscopy structure of isoproterenol-bound β1-AR (PDB code: 7JJO) and iperoxo-bound CHRM3 (PDB code: 8EA0) in the RCSB Protein Data Bank (PDB) database (http://www.rcsb.org; accessed on 12 August 2024, this study selected their agonists isoproterenol and iperoxo as ligand controls and guided the docking of other compounds with β1-AR and CHRM3. In addition, the sialogogue drug pilocarpine was used as a ligand control of CHRM3. The molecular docking operation was as follows. The 3D structures of β1-AR (7JJO) and CHRM3 (8EA0) were downloaded from the PDB database. The 2D structures of the active compounds in BZYQF and pilocarpine were downloaded from the databases of PubChem (https://pubchem.ncbi.nlm.nih.gov/; accessed on 12 August 2024), TCMSP (https://www.tcmsp-e.com/#/home; accessed on 12 August 2024), or ZINC (http://docking.org/; accessed on 12 August 2024). Autodock version 4.2.6 was used to remove the ligand molecule and conjugated G protein from 7JJO and 8EA0. The 2D structures of all compounds were imported into Discovery Studio 2019 (http://www.discoverystudio.net/; accessed on 12 August 2024) for ligand processing and selection of the optimal configuration. The optimized protein structures of 7JJO and 8EA0 were imported into Discovery Studio 2019 for batch docking with these preprocessed compounds. According to the structure of isoproterenol-bound β1-AR (7JJO), the coordinates of binding sites were set as x 133.53, y 132.95, and z 174.47, and the grid size was set as 32 in molecular docking for β1-AR. According to the structure of iperoxo-bound CHRM3 (8EA0), the coordinates of binding sites were set as x 138.11, y 121.69, and z 143.47, and the grid size was set as 49 in molecular docking for CHRM3. All the coordinates of binding sites and grid size in this study are shown in the Supplementary Materials. The CDOCKER module of Discovery Studio 2019 was used to analyze the interaction energy of β1-AR and CHRM3 with active compounds in BZYQF. CDOCKER interaction energy (kcal/mol) indicates the energy of the protein-compound interaction, where a lower value reflects that the compound is easier to combine with the protein. A value of −40 Kcal/mol suggests that the compound has the affinity to bind to the protein. Hence, the parameters (e.g., binding sites of amino acid residues and interactions) were obtained based on the docking results with CDOCKER interaction energy ≤ −40 Kcal/mol.

### 4.4. Induction of T2DM Rats and BZYQF Treatment

This study was authorized by the Laboratory Animal Ethics Committee of Guangdong Pharmaceutical University (Grant No. gdpulacspf2022203 and date of approval: 12 May 2023) and strictly abided by animal welfare ethics. In order to control the potential confounders among rats, such as age, gender and weight, male Sprague Dawley (SD) rats that were 7 weeks old, with a body weight of 190–210 g, were purchased from the Medical Experimental Animal Center of Guangdong Province. The production license number was SCXK (Guangdong) 2022–0002. T2DM was induced according to our previous method [27]. A brief summary of this method is as follows: after feeding for 3 days, SD rats were randomly divided into two groups of control (CON, N = 8) and disease (N = 22). The CON rats were provided basic feed, while the disease rats were provided high-sugar/fat feed. After feeding for 8 weeks, the disease rats were injected intraperitoneally with 35 mg/kg streptozotocin (STZ, Sigma, Saint Louis, MO, USA), and the CON rats were injected with an equivalent citrate buffer after fasting for 12 h. After STZ injection for 2 week, the disease rats were induced successfully when rats were found to have a fasting blood glucose (FBG) level ≥ 11.1 mmol/L and lower fasting blood insulin and HOMA-IS than CON rats in addition to polyphagia, polyuria, polydipsia, and weight loss. Then, 24 disease rats were randomly divided into the T2DM group (N = 8), the BZYQF group (N = 8), and the metformin hydrochloride group (MH, N = 6). According to our preliminary experimental results and references [28], the rats in the BZYQF group were intragastrically administrated BZYQF at a dose of 8 g/kg/d. This dose was calculated using the body surface area normalization method [50] according to the suggested human dose [51]. The rats in the MH group were intragastrically administered 210 mg/kg/d of metformin (batch no.ABT8108, Shiguibao Pharmaceutical Co., Ltd., Shanghai, China) in accordance with clinical dosing principles and our previous report [52]. The rats in the T2DM group and the CON group were intragastrically administered equivalent physiological saline. All rats were intragastrically administered for 11 weeks. In the entire process of the experiment, daily indicators, such as food intake, water intake, and body weight of rats, were recorded once a week. The experimental rats were bred in the Experimental Animal Center of Guangdong Pharmaceutical University. The feeding conditions were a room temperature of 20 ± 2 °C, a 12/12 h light/dark cycle, and a relative humidity of 60–70%.

### 4.5. Sample Collection and Processing

All samples were collected and processed according to our previous method [8,53]. At 9:00–11:00 a.m., after fasting for 12 h, the experimental rats were anesthetized with 1% pentobarbital sodium. While the rats were in a semiconscious state, basal saliva was collected from their oral cavity for 5 min using a microsampler. Then, the tip of the rat’s tongue was stimulated with 0.4 mol/L of citric acid filter paper (0.5 × 0.5 cm) for 30 s and stimulated saliva was immediately collected for 2.5min. This stimulated process was repeated once to obtain sufficient saliva, and the saliva collected during these 5 min was considered as stimulated saliva. After freezing at −20 °C overnight, the collected saliva samples were centrifuged at 10,000× *g* for 5 min at 4 °C, and the supernatant was stored at −80 °C. The blood samples were collected from the eye sockets of rats. After standing at 4 °C for 2 h, the blood sample was centrifuged at 1000× *g* for 15 min to collect serum and stored in a −80 °C refrigerator. At the end of the animal experiment, all rats were euthanized via the abdominal aortic drainage method, and the rats’ PG and SMG were extracted within 15 min. Some of tissue samples were fixed in 4% paraformaldehyde, and the remaining tissues were stored at −80 °C.

### 4.6. Determination of Biochemical Indicators in Saliva, Salivary Glands, and Serum

According to our previous method [8], the sAA activity (U/mL) was determined using the kinetic reaction assay kit (α-AMY kit, Cat. No. 105-000475-00, Mindray, Shenzhen, China) on the BS-180 automatic biochemical analyzer (Mindray, Shenzhen, China). The total protein content (mg/mL) was determined on NanoDrop microvolume spectrophotometers (ThermoFisher, Waltham, MA, USA) by the UV absorption method, and bovine serum albumin was used to obtain standard curves. All procedures were conducted strictly in accordance with the reagents manual and instrument operation instructions. The saliva volume (μL) and collection time (min) were used to calculate salivary flow rate (μL/min). Salivary protein secretion rate (mg/min) was calculated using total protein concentration (mg/mL) and salivary flow rate (μL/min). The sAA-specific activity (U/mg) was calculated using sAA activity (U/mL) and total protein concentration (mg/mL). The difference (delta) value of all these salivary indicators was calculated using their values in basal and stimulated saliva.

The tissue samples of the PG and SMG that were stored in the −80 °C refrigerator were then used to prepare 10% tissue homogenate with pre-cooling saline using an electric homogenizer. After centrifugation at 10,000× *g* for 10 min at 4 °C, the supernatant was used to measure biochemical indicators in salivary glands. Total protein content (mg/mL) was determined on NanoDrop microvolume spectrophotometers. The contents of glucose, triglyceride (TG), and total cholesterol (TC) in salivary glands and serum and the sAA activity in salivary glands were determined with a commercially available kit (glucose kit, Cat. No. 105-000949-00; TG kit, Cat. No. 105-000449-00; TC kit, Cat. No. 105-000448-00; α-AMY kit, Cat. No. 105-000475-00; Mindray, Seoul, The Republic of Korea) on the BS-180 automatic biochemical analyzer. The contents of acetylcholine (Ach, Cat. No. A105-2(1)-1, Jiancheng Biogengineering ins., Nanjing, China) in the salivary glands and serum were measured on an Elx800 microplate reader (BioTek, Winooski, VT, USA). The content of fasting serum insulin (FINS, Cat. No. ER1113, Fine Biotech Co., Ltd., Wuhan, China) was measured on an Elx800 microplate reader (BioTek, Winooski, VT, USA) using an enzyme-linked immunosorbent assay (ELISA). All operations were strictly carried out in accordance with the instructions of the reagent kit and the usage guide of instrument.

### 4.7. Hematoxylin and Eosin and Immunohistochemical Staining

The tissue specimens of the PG and SMG were extracted from 4% paraformaldehyde, subsequently dehydrated using gradient ethanol, and embedded in paraffin using a Heated Paraffin Embedding Station (HistoCore Arcadia H, Leica, Nussloch, Germany). The paraffin samples were then cut into 4–5 μm thick slices using a paraffin microtome (Leica RM2235, Nussloch, Germany). Some sliced specimens were deparaffinized with xylene and rehydrated in graded ethanol. Finally, these specimens were stained with a hematoxylin and eosin (HE) staining kit (Cat. No. G1120, Solarbio Technology Co., Ltd., Beijing, China) and coverslipped with a mounting reagent (Neutral balsam, #S3006, Shanghai Specimen and Model Factory, Shanghai, China). Images were captured using an optical microscope (Leica DM500, Nussloch, Germany). Other slices were deparaffinized and subjected to antigen repair, followed by blocking with goat serum (Cat. No. AR0009, Boster Biological Technology Co., Ltd., Wuhan, China) for 1 h. These slices were then incubated with the primary antibody against sAA (Cat. No. A12967, 1:250, Boster, Wuhan, China) and the secondary antibody (Cat. No. GP2403018, Servicebio Technology Co., Ltd., Wuhan, China). Finally, these specimens were developed with the DAB substrate kit (Cat. No. PA110, Tiangen Biotech Co., Ltd., Beijing, China) and counterstained with hematoxylin. Images were captured under different fields of view and magnifications using an optical microscope (Leica DM500, Nussloch, Germany). HE and immunohistochemical staining results were quantified using ImageJ v1.8.0 software (National Institutes of Health, Bethesda, MD, USA). The inflammatory infiltration scoring criteria were as follows: no inflammation (0 points); light inflammation (1 point); moderate inflammation (2 points); severe inflammation (3 points) [54].

### 4.8. Real-Time Quantitative PCR

Total RNA was extracted from the PG and SMG tissues using RNAiso Plus (Cat. No. 9109, Takara Bio Inc., Kusatsu, Japan). The extraction quality of the total RNA was detected using a NanoDrop Lite spectrophotometer (Thermo Fisher Scientific, Inc., Walham, MA, USA). Total RNA with an A_260_/A_280_ value of 1.9–2.1 was used for reverse transcription into cDNA using the FastKing gDNA Dispelling RT SuperMix (Cat. No. KR118, Tiangen Biotech Co., Ltd., Beijing, China). Real-time quantitative PCR (RT-qPCR) was performed using 2 × Color SYBR Green qPCR Master Mix (Cat. No. A0012-R2, EZBioscience, San Diego, CA, USA) on the CFX Connect Real-Time System (Bio-Rad, Hercules, CA, USA). The primers and product sizes used in RT-qPCR are listed in Table 6. The PCR thermal cycle was as follows: initial denaturation at 95 °C for 5 min, followed by 40 cycles at 95 °C for 10 sec and 60 °C for 30 sec. β-actin was used as the reference gene, and the relative expression of target genes was quantified using the 2^−ΔΔCt^ method. The PCR of every gene was repeated at least three times. All operations were carried out according to the instructions of the reagent kit and instrument.

### 4.9. Western Blot

Frozen tissue samples from the PG and SMG were homogenized in RIPA lysis buffer (Cat. No. P0013B, Beyotime, Shanghai, China) with 1 mM PMSF (Cat. No. P0100, Solarbio, Beijing, China) on ice for 30 min. After centrifugation at 10,000× *g* for 10 min at 4 °C, the total protein concentration was measured using a NanoDrop Lite spectrophotometer (Thermo Fisher Scientific, Waltham, MA, USA). An amount of 35 μg of total protein was separated by 5–10% SDS-PAGE on a Mini-Protean Tetra Cell (Bio-Rad, USA). The separated proteins were transferred onto PVDF membranes on a Mini Trans-Blot Electrophoretic Transfer Cell (Bio-Rad, USA) and blocked with 5% skim milk for 2 h at room temperature. The protein blots were then incubated with primary antibodies against β1-AR (Cat. No. P08588, 1:1300, Biorbyt Ltd., Cambridge, UK), CHRM3 (Cat. No. DF2781, 1:700, Affinity Biosciences, Jiangsu, China), AMY1 (sAA, Cat. No. A12967, 1:800, Boster, Wuhan, China), and β-actin (Cat. No. R23613, 1:7000, Zen-BioScience, Chengdu, China) overnight at 4 °C. After washing with PBST three times, the protein blots were incubated with secondary antibodies (Cat. No. BS13278, 1:6000, Bioworld Technology Inc., Nanjing, China) for 1 h at room temperature. After washing with PBST three times, the protein signal was detected using an ECL kit (Cat. No. P0018FS, Beyotime, Shanghai, China) and captured by GeneGnome XQR gel imaging systems (Syngene, Cambridge, UK). Western blot results were quantified using ImageJ v1.8.0 software (National Institutes of Health, Bethesda, MD, USA).

### 4.10. Statistical Analysis

Data analysis and figure generation were performed using the GraphPad Prism v 9.4.0 software (GraphPad Software Inc., San Diego, CA, USA). One-way ANOVA was conducted to analyze the difference among the multiple groups, and Student’s *t*-test was performed to analyze the differences between two groups. The difference effect in salivary parameters between the rats of the T2DM group and the CON group and between the BZYQF group and T2DM group was estimated by Cohen’s d value. A value of *p* < 0.05 was considered statistically significant. The results are expressed as mean ± standard deviation.

## 5. Conclusions

Through network pharmacology analysis and molecular docking, the present study revealed that the compounds Jaranol, Mairin, quercetin, Licoricone, isorhamnetin, kaempferol, Licochalcone B, naringenin, Nicotinic acid, and Formononetin in BZYQF were able to improve saliva secretion by targeting signaling molecules in the salivary secretion pathway. Subsequently, we constructed a T2DM rat model to evaluate the efficacy of BZYQF. The results showed that BZYQF can alleviate T2DM symptoms and promote the secretion of basal and stimulated saliva in T2DM rats. Further mechanistic studies demonstrated that BZYQF can reduce glucose and lipid accumulation, enhance acetylcholine content, and improve pathological lesions and inflammation, as well as increasing the expression of signaling molecules—including PKA, IP3R, β1-AR, AQP5, CHRM3, and AMY1—in the salivary secretion pathway in the PG and SMG of T2DM rats. These results suggest that BZYQF can alleviate T2DM-caused hyposalivation through activation of the salivary secretion pathway in the PG and SMG of T2DM rats. This study provides a theoretical basis for the use of BZYQF in treating T2DM and T2DM-caused hyposalivation. The results of this study need to be validated through human trials in order to be applied more effectively in clinical practice. Furthermore, the binding sites and affinity toward β1-AR and CHRM3 of these screened compounds still need to be validated through experiments such as site-directed mutagenesis or receptor-binding assays. The molecular mechanism of BZYQF’s treatment of T2DM-caused hyposalivation also needs to be further validated through in vitro experiments. These needs will guide the direction of future research.

## Figures and Tables

**Table 1:** The sources and classification of validated compounds targeting β1-AR and CHRM3 with favorable ADMET properties in BZYQF.

Compounds	Class I	Sources	Compounds	Class I	Sources
isorhamnetin	Flavonoids	CaiHu, HuangQi, GanCao	Mairin	Terpenoids	HuangQi, GanCao
kaempferol	Flavonoids	CaiHu, HuangQi, GanCao	Licoricone	Flavonoids	GanCao
Licochalcone B	Flavonoids	GanCao	petunidin	Flavonoids	CaiHu
naringenin	Flavonoids	ChenPi	Atractylenolide I	Terpenoids	BaiZhu
Nicotinic Acid	Others (Vitamin)	DangGui	Atractylenolide II	Terpenoids	DangShen
quercetin	Flavonoids	CaiHu, HuangQi, GanCao	Formononetin	Flavonoids	GanCao
Jaranol	Flavonoids	HuangQi			

**Table 2:** The serum biochemical indexes in experimental rats.

Group	N	Glucose (mmol/L)	TC (mmol/L)	TG (mmol/L)	Ach (μg/mL)	FINS (μU/mL)	HOMA-IS
CON	8	5.93 ± 0.53 ***	1.89 ± 0.20 **	0.71 ± 0.17 **	21.50 ± 1.87 **	13.71 ± 1.79 ***	120.27 ± 43.84 ***
T2DM	7	23.22 ± 2.54	4.85 ± 2.62	5.39 ± 3.71	16.47 ± 2.40	5.37 ± 1.12	5.54 ± 1.39
BZYQF	8	19.81 ± 1.08 **	2.72 ± 0.81 *	2.30 ± 1.11 *	31.18 ± 3.76 ***	10.44 ± 3.68 **	12.99 ± 4.90 **
MH	6	19.06 ± 1.66 **	3.43 ± 1.25	1.75 ± 0.70 *	31.02 ± 3.98 ***	**-**	**-**

* *p* < 0.05, ** *p* < 0.01, and *** *p* < 0.001, compared with T2DM rats.

**Table 3:** The delta value of the salivary parameters between basal and stimulated saliva in experimental rats.

Group	N	Salivary Flow Rate	Total Protein Content	Protein Secretion Rate	sAA Activity	sAA Specific Activity
CON	8	26.73 ± 13.07 *	0.77 ± 0.43 *	0.04 ± 0.11	3.32 ± 1.26 ***	0.94 ± 0.82 *
T2DM	8	15.83 ± 3.86	0.19 ± 0.59	0.10 ± 0.17	0.47 ± 0.54	0.23 ± 0.33
BZYQF	8	29.98 ± 15.06 *	0.74 ± 0.37 *	0.05 ± 0.11	2.63 ± 0.93 ***	0.74 ± 0.52 *
MH	6	30.94 ± 14.57 *	0.56 ± 0.29	0.10 ± 0.05	1.72 ± 1.30 *	0.15 ± 0.29

* *p* < 0.05 and *** *p* < 0.001, compared with T2DM rats.

**Table 4:** Biochemical parameters of PG and SMG in experimental rats.

	CON (N = 8)		T2DM (N = 8)		BZYQF (N = 8)	
	PG	SMG	PG	SMG	PG	SMG
Glucose (mmol/L)	0.12 ± 0.04 ***	0.05 ± 0.01 ^##^	0.51 ± 0.17	0.25 ± 0.16	0.35 ± 0.09 *	0.10 ± 0.04 ^#^
TC (mmol/L)	0.21 ± 0.07 **	0.09 ± 0.05	0.36 ± 0.07	0.13 ± 0.02	0.28 ± 0.07	0.11 ± 0.03
TG (mmol/L)	0.75 ± 0.34 ***	0.28 ± 0.09	2.48 ± 0.48	0.30 ± 0.15	1.40 ± 1.10 *	0.22 ± 0.08
sAA activity (U/mL)	1.19 ± 0.16 ***	2.00 ± 0.15 ^###^	0.52 ± 0.40	1.05 ± 0.24	1.11 ± 0.31 **	1.81 ± 0.56 ^##^
Ach (mg/g)	1.30 ± 0.16 ***	1.48 ± 0.16 ^###^	0.78 ± 0.07	0.98 ± 0.10	1.07 ± 0.16 **	1.21 ± 0.12 ^##^
Total protein (mg/mL)	4.91 ± 0.45	8.60 ± 0.49	4.75 ± 0.56	8.55 ± 0.38	4.88 ± 0.44	8.97 ± 0.48

* *p* < 0.05, ** *p* < 0.01, and *** *p* < 0.001, compared with PG of T2DM rats; ^#^ *p* < 0.05, ^##^ *p* < 0.01, and ^###^ *p* < 0.001, compared with SMG of T2DM rats.

**Table 5:** The Chinese herbs and dosage of BZYQF.

Chinese Name	Latin Name	English Name	Part Used	Dosage
HuangQi	*Astragalus membranaceus* (Fisch.) Bunge	Astragali radix	Root	200 g
DangShen	*Codonopsis pilosula* (Franch.) Nannf	Codonopsis radix	Root	60 g
GanCao	*Glycyrhiza uralensis* Fisch.	Glycyrrhizae radix et rhizoma Praeparata cum melle	Root	100 g
BaiZhu	*Atractylodes macrocephala* Koidz.	Atractylodis macrocephalae Rhizoma	Root	60 g
DangGui	*Angelica sinensis* [Oliv.] Diels.	Angelicae sinensis radix	Root	60 g
ChenPi	*Citrus reticulata* Blanco.	Citri reticulatae pericarpium	Pericarp	60 g
ShengMa	*Cimicifuga heracleifolia* Kom	Cimicifugae rhizoma	Root	60 g
ChaiHu	*Bupleurum chinense* DC.	Bupleuri radix	Root	60 g
ShengJiang	*Zingiber offcinale* Roscoe.	Rhizoma zingiberis recens	Root	20 g
DaZao	*Ziziphus jujuba* Mill.	Chinese-date		40 g

The plant name has been checked according to https://wfoplantlist.org/ (accessed on 2 May 2024).

**Table 6:** Sequences of primers and product size used in RT-qPCR.

Gene	Forward Primer (5′→3′)	Reverse Primer (5′→3′)	Product Size
*IL-6*	AGGAGTGGCTAAGGACCAAGACC	TGCCGAGTAGACCTCATAGTGACC	85 bp
*TNF-α*	GCATGATCCGAGATGTGGAACTGG	CGCCACGAGCAGGAATGAGAAG	113 bp
*PKA*	GGACAAGCAGAAGGTGGTGAAGC	ACCAGGCACGTACTCCATGACC	154 bp
*IP3R*	CCGTACAAGTACGTGCTGCGTCTC	GCCGATCTGAGACTGCATGACGC	146 bp
*β1-AR*	CTCATCGTGCTGCTCATCGTAGTG	GATGAAGAGGTTGGTGAGCGTCTG	93 bp
*AQP5*	CAACAACACAACGCCTGGCAAG	AGAGTCGGTGGAGGAGAAGATGC	88 bp
*CHRM3*	CGGTCGCTGTCACTTCTGGTTC	CGCTGCTGCTGTGGTCTTGG	80 bp
*AMY1*	CTGGTTGATACAAGTTGATGAGAG	TATCAACCCAGCGCCACTC	420 bp
*β-actin*	GGAGATTACTGCCCTGGCTCCTA	GACTCATCGTACTCCTGCTTGCTG	150 bp

*IL-6*, interleukin-6; *TNF*, tumor necrosis factor; *PKA*, protein kinase A; *IP3R*, Inositol 1,4,5-Trisphosphate Receptor; *β1-AR*, β1 adrenergic receptor; *AQP5*, aquaporin-5; *CHRM3*, cholinergic receptor; *AMY1*, salivary alpha-amylase (sAA); *β*-actin was used as the reference gene.

## Data Availability

The original contributions presented in this study are included in the article/Supplementary Materials. Further inquiries can be directed to the corresponding author.

## Supplementary Materials

The following supporting information can be downloaded at https://www.mdpi.com/article/10.3390/ph18030377/s1: Figure S1: MRM_detection_of_multimodal_maps-N; Figure S2: MRM_detection_of_multimodal_maps-P; Table S1: All compounds were identified in BZYQF by UPLC-MSMS; Table S2: The coordinates of binding sites and grid size in this study.

## Abbreviations

The following abbreviations are used in this manuscript:

BZYQF	BuZhong YiQi Formula
T2DM	type 2 diabetes mellitus
PG	parotid gland
SMG	submandibular gland
SLG	sublingual gland
TCM	Traditional Chinese Medicine
GO	Gene Ontology
KEGG	Kyoto Encyclopedia of Genes and Genomes
sAA and AMY1	salivary alpha-amylase
IL-6	interleukin-6
TNF	tumor necrosis factor
PKA	protein kinase A
IP3R	inositol 1,4,5-trisphosphate receptor
β1-AR	β1 adrenergic receptor
AQP5	aquaporin-5
CHRM3	cholinergic receptor
FBG	fasting blood glucose
TC	total cholesterol
TG	triglyceride
Ach	acetylcholine
FINS	fasting serum insulin
MH	metformin hydrochloride
