# Supplementary figures and images for: BuZhong YiQi Formula Alleviates Diabetes-Caused Hyposalivation by Activating Salivary Secretion Pathway in the Parotid and Submandibular Glands of Rats
Source: Pharmaceuticals (Basel). 2025 Mar 6;18(3):377. doi: 10.3390/ph18030377 (PMC11944908; doi:10.3390/ph18030377)

■ XIC of -MRM (1906 pairs): 198.000/181.000 amu Expected RT: 2.6 ID: mws0923\_N from Sample 2 (T2360676a\_N) of MWXS-23-777-01-a\_1\_W...

Max. 2.9e5 cps.

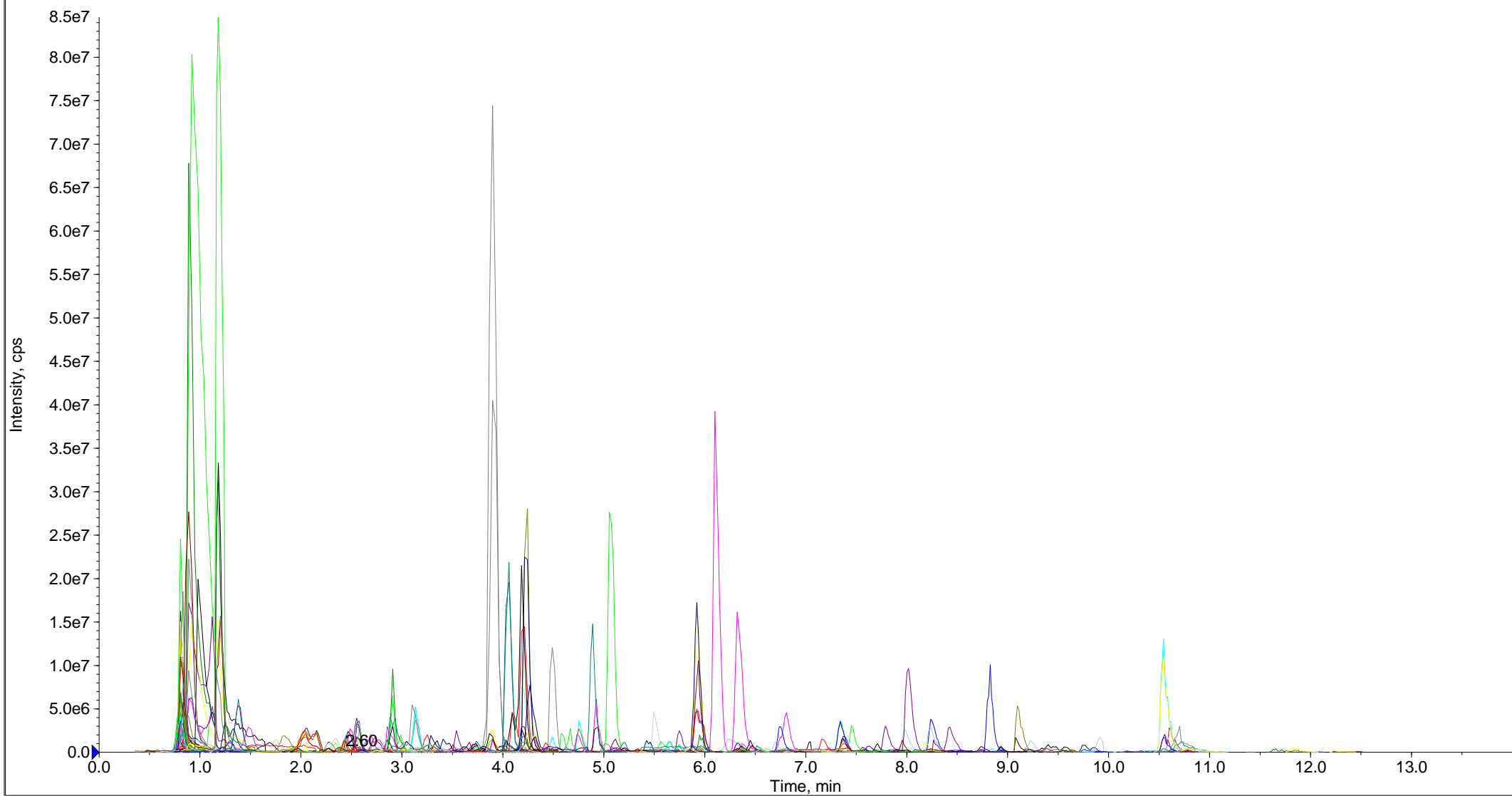

Supplement: Supplementary file 1 [file pharmaceuticals-18-00377-s001.zip › Supplementary Materials/Figure S1-MRM_detection_of_multimodal_maps-N.pdf]

■ XIC of +MRM (2237 pairs): 200.000/154.000 amu Expected RT: 2.5 ID: mws0923\_P from Sample 1 (T2360676a\_P) of MWXS-23-777-01-a\_1\_W...

Max. 1.2e7 cps.

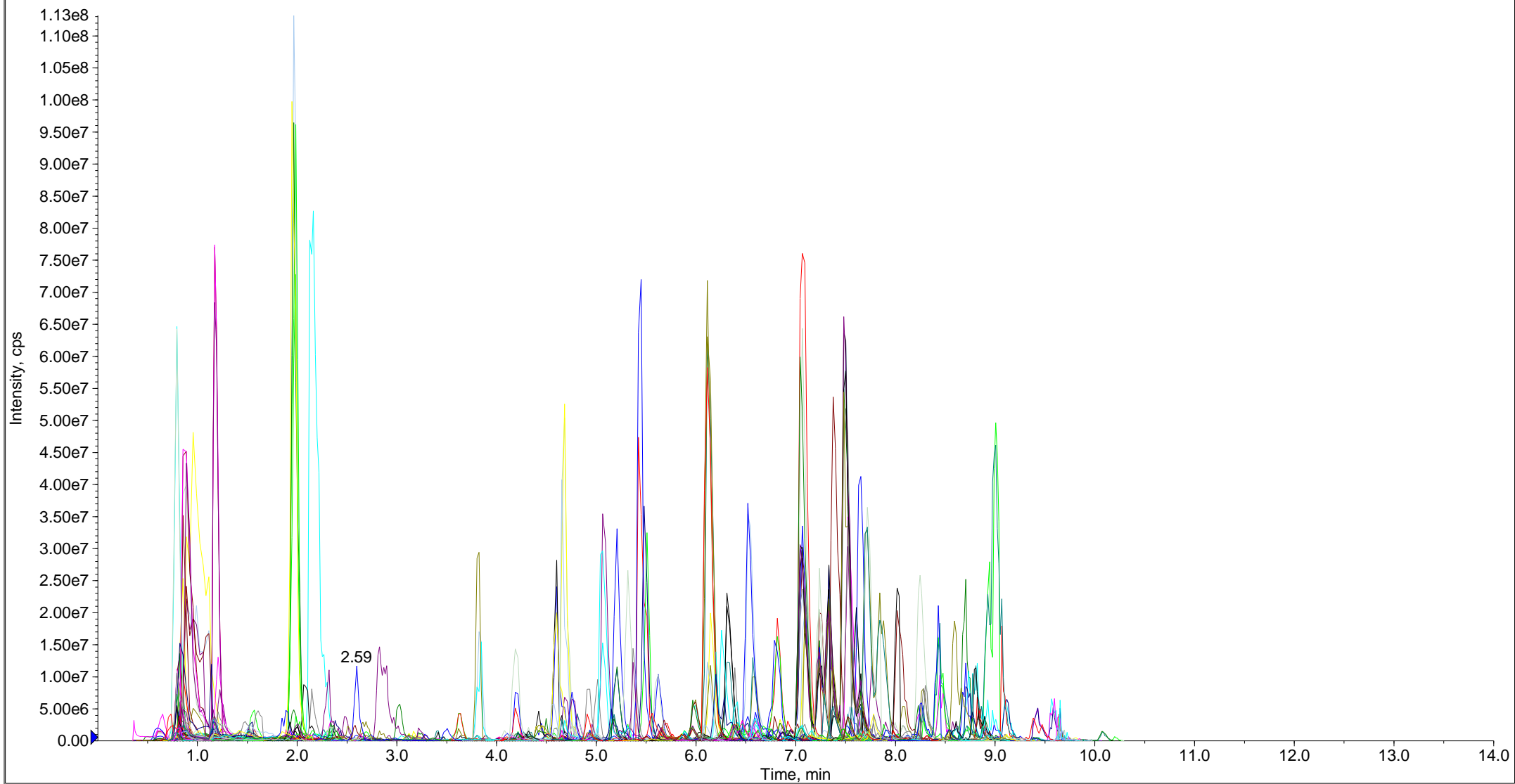

Supplement: Supplementary file 1 [file pharmaceuticals-18-00377-s001.zip › Supplementary Materials/Figure S2-MRM_detection_of_multimodal_maps-P.pdf]
